# The impact of helicobacter pylori eradication on platelet counts of adult patients with idiopathic thrombocytopenic purpura

**DOI:** 10.1186/s12878-018-0119-y

**Published:** 2018-09-20

**Authors:** Sara Aljarad, Ahmad Alhamid, Ahmad Sankari Tarabishi, Ameen Suliman, Ziad Aljarad

**Affiliations:** 1 Department of Hematology, Al Mouwasat University Hospital, Damascus, Syria; 2 Department of Gastroenterology, Aleppo University Hospital, Aleppo, Syria; 3 Medical student, Faculty of Medicine, University of Aleppo, Aleppo, Syria

**Keywords:** Idiopathic thrombocytopenic purpura, Helicobacter pylori, Platelet disorders

## Abstract

**Background:**

Idiopathic (immune) thrombocytopenic purpura (ITP) is an acquired disorder characterized by autoantibodies against platelet membrane antigens. Several studies found an association between Helicobacter Pylori infection and the incidence of ITP. So far, It is still unclear whether *H. pylori* eradication will increase platelet counts in adult ITP patients. We conduct this study to investigate platelet recovery in ITP patients after *H. pylori* eradication.

**Methods:**

This is a prospective study. The diagnostic criterion for Idiopathic thrombocytopenic purpura is: isolated thrombocytopenia, with no evidence of any underlying causes like drugs, TTP, SLE, hepatitis, HIV,CLL and… etc. We examined blood smears of all patients. We have diagnosed Helicobacter pylori infection by histological examination of several biopsies obtained from stomach and duodenum by esophagogastroduodenoscopy (EGD). If EGD was not applicable due to patient’s poor situation or platelet count, H.pylori infection was diagnosed by the positivity of serum antibodies or respiratory urease test. We treated infected patients with triple therapy (omeprazole 40 mg once daily, amoxicillin 1000 mg twice daily and clarithromycin 500 mg twice daily) for 14 days. Uninfected patients did not receive any treatment. We did platelet quantification at the beginning of the study, at the end of the first month, at the end of the third month and at the end of the sixth month.

**Results:**

This study involved 50 patients with chronic ITP, 29 males (58%) and 21 females (42%). Participants ages range between18 and 51 years (mean age = 28.60 years). We diagnosed *H. pylori* in 36 patients (72%), who were treated with triple therapy. At the end of the sixth month, 10 of them (27.77%) showed complete response, and 18 of them (50%) showed partial response. The 14 uninfected patients, who did not receive any treatment, did not show neither complete nor partial response. Patient sex and age were not associated with achieving response, while baseline platelet count and H.pylori infection did.

**Conclusion:**

Helicobacter pylori eradication significantly increases platelet counts in adult ITP patients.

## Background

Idiopathic (immune) thrombocytopenic purpura (ITP) is an acquired disorder characterized by autoantibodies against platelet membrane antigens [1].

There are considerable differences in the clinical manifestations among ITP patients. The onset may be acute and sudden or may be insidious, and may result in significant mortality and morbidity. Patients may be asymptomatic, and symptoms in symptomatic patients range from easy bruising to severe bleeding [1].

Incidence rate of ITP is about 50–100 new cases per million per year, half of them are children. At least 70% of cases diagnosed in childhood will recover completely within six months, even without treatment [2, 3]. A third of the remaining chronic cases will completely recover during follow-up [4, 5], another third will end up with only mild thrombocytopenia (platelet count above 50 × 10^9^/L) [6].

Thrombocytopenia Purpura is usually chronic in adults [7], and the probability of complete remission is 20–40. Male to female ratio in the adult group clearly differs in most age groups (children approximately have equal incidence in both sexes. The average age at diagnosis in adults is 56–60 years [8].

Helicobacter pylori (*H. pylori*) is a gram-negative microaerophilic bacterium that colonizes in the stomach. *H. pylori* is implicated in the development of active chronic gastritis, gastric ulcers, and duodenal ulcers.

*H. pylori* is a cofactor in the development of both gastric adenocarcinoma and mucosa-associated lymphoid tissue lymphoma. Recently, It has been discovered that *H. pylori* is implicated in various autoimmune disorders, including pernicious anemia and idiopathic thrombocytopenic purpura (ITP) [9], linked to the development of peptic ulcers in stomach and gastric carcinoma. Approximately 50% of the world’s population are infected with *H. pylori*, making it the most prevalent bacterial infections in the world. Its prevalence is greater in low-income countries than in developed ones [10]. The exact route of infection is still unknown, but fecal-oral and oral-oral routes seem to be the most likely [10].

No enough evidence is available to determine the impact of H.pylori eradication on platelet count in ITP patients. We also have not found in the medical literature a study in our region about the prevalence of *H. pylori* infection among ITP patients, and the effect of *H. pylori* eradication on ITP patients in our country, as related studies differ in their results from country to another. The objective of this research is to:

1. Determine the prevalence of *H. pylori* infection in ITP patients.

2. Evaluate the response of ITP patients to *H. pylori* eradication.

## Methods

This prospective study was conducted at the University of Damascus, Division of Hematology at Al-Mouwasat University Hospital and Al Assad University Hospital- Department of Internal Medicine, between October 2016 and October 2017. The study included all adult patients of both sexes, who were diagnosed with ITP according to the American Society of Hematology as follows:

- General platelet count is less than 100 × 10^9^/L.
- Exclusion of the secondary causes of thrombocytopenia (e.g. drugs, hepatitis C virus, Human Immunodeficiency virus, pseudothrombocytpenia, malignancies). We examined blood smears of all patients.

### Exclusion criteria

- Patients younger than 14 years old.
- Patients with life-threatening bleeding or an active hemorrhage requiring immunosuppression or other therapeutic options to increase platelet count.
- Patients with secondary causes of thrombocytopenia, including any drugs that are suspected of developing thrombocytopenia.
- Taking antibiotics, proton pump inhibitors or H2 blockers a month before screening for *H. pylori*, because these lead to false negatives.
- Any previous *H. pylori* eradication program.

If platelet count and patient status allowed, We diagnosed H.pylori infection was by histological examination of different biopsies obtained from different areas of stomach and duodenum (including the gastric antrum) via esophagogastroduodenoscopy (EGD). IF EGD is not applicable, We diagnosed *H. pylori* infection by the positivity of serum antibodies or urease breath test. We first did serological testing. If the serology was positive, we considered the patient infected. If serology was negative, we did urease breath test. Infected patients were then given triple therapy for H.pylori (omeprazole 40 mg once daily, amoxicillin 1000 mg twice daily, clarithromycin 500 mg twice daily) for 14 days. They were not given any additional ITP treatment to raise platelet count. Patients who were not infected with H.Pylori did not receive any treatment that aimed to increase platelet count during the follow-up period. This method helps to ensure that differences in outcomes are more likely to result from H.pylori eradication rather than other confounding treatments.

Patients had chronic ITP, which means that patients have taken several treatment lines like corticosteroids, splenectomy, IVIG and danazol before detecting HP but without. In addition, no life-threatening bleeding happened during the follow-up period. So, we did not give any therapeutic agent that may causes rising platelet count to avoid confounding differences in results between Hp positive and Hp negative patients.

Patients were followed up for 6 months. We did baseline platelet quantification, at the end of the first month, at the end of the third month and at the end of the sixth month.

No loss to follow-up or data missing happened.

### Response criteria

As several similar studied did, We adopted the following definitions of response:

Complete response: Platelet count of more than 150 × 10^9^/L (within the normal range).

Partial response: Elevation of Platelet count from 50 × 10^9^/L to 50 × 10^9^/L, or twice the baseline platelet count.

### Statistical study

We performed statistical analyses with SPSS (Version 22.0; SPSS Inc.: Chicago, IL, USA). We used P- value to evaluate statistical significance of differences between groups. The level of significance is *P* < 0.05. Categorical variables were described using frequencies and percentages. Numerical variables were described using (mean or median standard deviation). In order to examine the significance of difference between groups, we conducted Chi-square test and Fisher exact test for categorical variables, and Student T test and the Mann-Whitney U test for numerical variables.

## Results

The final sample of the study included 50 patients, 29 of them were males (58%), and 21 were females (42%). Participants ages were between 18 and 51 years (mean age = 28.60 years), with a standard deviation of 8.75 years.

The prevalence of H.pylori infection was as following:

Thirty six patients (72%) were diagnosed with *H. pylori* infection.

**Fig. 1:**
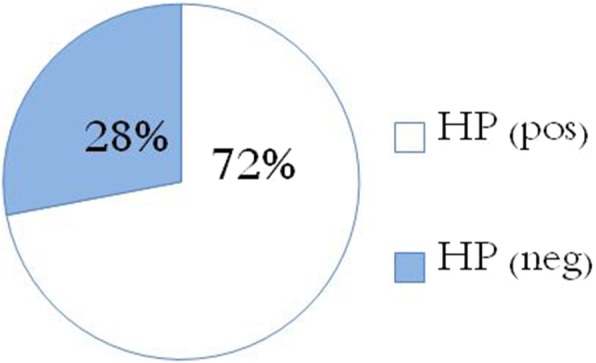
H.pylori Infection Among ITP Patients

*H. pylori* infection was diagnosed by the positivity of both respiratory urease test or serum antibodies in 20 patients (55.56%), and by histological examination of gastric and duodenal biopsies in 16 patients (44.44%), as shown in (Table 1) and (Fig. 1).

**Table 1:** Prevalence of infection with H. Pylori and diagnostic methods used

H.pylori infection:	Number of cases (%):	Diagnostic method used:
*H. pylori* positive (Hp^+^) Patients	36 (72%)	• Positivity of serum antibodies or urease breath test in 20 patients (55.56%). • Histological examination of gastric and duodenal biopsies obtained by EGD in 16 patients (44.44%).
H. Pylori negative (Hp^−^) Patients	14 (28%)	• The Respiratory Urease test and serum antibodies in 4 patients (28.5%). • Histological examination of gastrointestinal lesions from the gastrointestinal tract in 10 patients (71.5%).

### Platelet counts at the beginning of the study

At the beginning of the study, platelet counts in Hp^+^ Patients ranged from 22 × 10^9^/L to 88 × 10^9^/L, and the mean value was 46.25 × 10^9^/L (SD = 17.724). Platelet counts in Hp^−^ Patients ranged from 12 × 10^9^/L to 42 × 10^9^/L and the mean value was 25.21 × 10^9^/L (SD = 8.469). Independent sample T test shows that the mean platelet count of Hp ^−^ patients is significantly less than the mean platelet count of the Hp^+^ Patients (P (0.001). Table 2).

**Table 2:** Platelet Count during the study

Groups		Range of platelet counts (10^9^/L)	The mean value	Standard deviation	*P* value
Platelet Count at the beginning of the study	HP (+)	22–88	46.25	17.724	0.001
	HP (−)	12–42	25.21	8.469	
Platelet counts at the end of the first month	HP (+)	17–215	67.94	39.51	0.001
	HP (−)	21–36	28.28	4.71	
Platelet counts at the end of the third month	HP (+)	22–357	112.13	84.06	0.001
	HP (−)	15–42	28.42	6.489	
Platelet counts at the end of the sixth month:	HP (+)	15–212	98.66	59.54	0.001
	HP (−)	15–41	26.42	7.50	
difference in platelet counts between the end of the sixth month and baseline platelet count (platelet count at the end of the sixth month – baseline platelet count)	HP (+)	(− 37)-(187)	52.42	37.66	0.001
	HP (−)	(−27) –(19)	1.21	4.63	

### Platelet counts at the end of the first month

In Hp^+^ patients, platelet counts ranged from 17 × 10^9^/L to 215 × 10^9^/L, and the mean value was 67.94 × 10^9^/L (SD = 39.51). Platelet count increased in 23 patients (63.88%) compared to baseline counts, and decreased in 13 patients (36.12%). Only two patients (5.55%) achieved complete response (platelet count 150 × 10^9^/L), and 10 patients (27.77%) achieved partial response (platelet count elevation from to 50 × 10^9^/L, or twice the baseline platelet count). Overall response achieved in 12 patients (33.33%).

In Hp^−^ patients, platelet counts ranged from 21 × 10^9^/L to 36 × 10^9^/L, and the mean value was 28.28 × 10^9^/L (SD = 4.71). Independent sample T test shows that the mean platelet count at the end of the first month of the study in Hp ^−^ patients is significantly less than the mean platelet count of the Hp^+^ Patients (P (0.001) Table 2). Platelet count slightly increased in 6 patients (42.85%), but they did not achieve neither complete nor partial response.

### Platelet counts at the end of the third month

In Hp^+^ patients, platelet counts ranged from 22 × 10^9^/L to 357 × 10^9^/L, and the mean value was 112.13 × 10^9^/L (SD = 84.06). Platelet count increased in 27 patients (75%) compared to baseline counts, and decreased in 9 patients (25%). 8 patients (22.22%) achieved complete response, and 16 patients (44.44%) achieved partial response. Overall response achieved in 26 patients (72.22%).

In Hp^−^ patients, platelet counts ranged from 15 × 10^9^/L to 42 × 10^9^/L, and the mean value was 28.42 × 10^9^/L (SD = 6.489). Independent sample T test shows that the mean platelet count at the end of the third month of the study in Hp ^−^ patients is significantly less than the mean platelet count of the Hp^+^ Patients (P (0.001 Table 2)). Platelet count increased in 8 patients (57.15%), but they did not achieve neither complete nor partial response.

### Platelet counts at the end of the sixth month

In Hp^+^ patients, platelet counts ranged from 25 × 10^9^/L to 212 × 10^9^/L, and the mean value was 98.66 × 10^9^/L (SD = 59.54). Platelet count increased in 34 patients (94.44%) compared to baseline counts, and decreased in two patients (5.56%). 10 patients (27.77%) achieved complete response, and 18 patients (50%) achieved partial response. Overall response achieved in 28 patients (77.77%).

In Hp^−^ patients, platelet counts ranged from 15 × 10^9^/L to 41 × 10^9^/L, and the mean value was 28.42 × 10^9^/L. Independent sample T test shows that the mean platelet count at the end of the sixth month of the study in Hp ^−^ patients is significantly less than the mean platelet count of the Hp^+^ Patients (P (0.001) Table 2). Platelet count increased in 5 patients (25.71%) compared to baseline, but they did not achieve neither complete nor partial response.

We notice a slight decrease in the mean platelet count of Hp^+^ group at the end of the sixth month compared to that of the third month, so do the difference in means between Hp^+^ and Hp^−^ groups. But the mean platelet count of Hp^+^ group at the end of the sixth month is still greater than that at the beginning of the study and at the end of the first month, so do the difference in means between Hp^+^ and Hp^−^ groups .

Mean platelet count in Hp^+^ group at the end of the sixth month is still greater than the mean platelet count of Hp^−^ group at all stages. Furthermore, number of responding patients increased at the end of the sixth month.

**Fig. 2:**
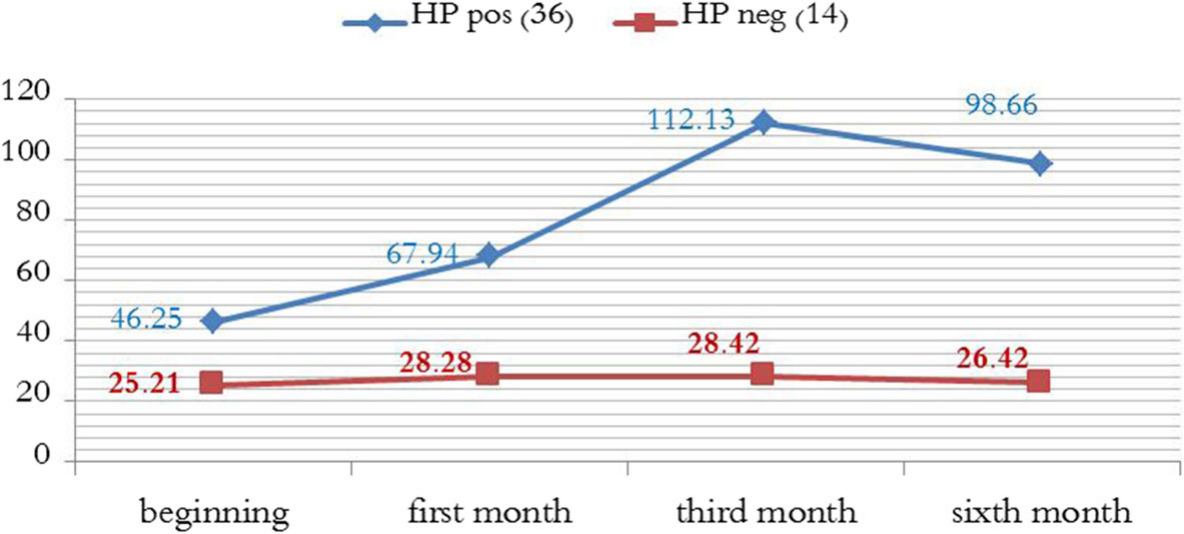
The mean values ​​of platelet counts in the two groups of *H. pylori* were changed during follow-up periods

Figure 2 shows the changes in mean platelet count in Hp^+^ and Hp^−^ groups.

**Fig. 3:**
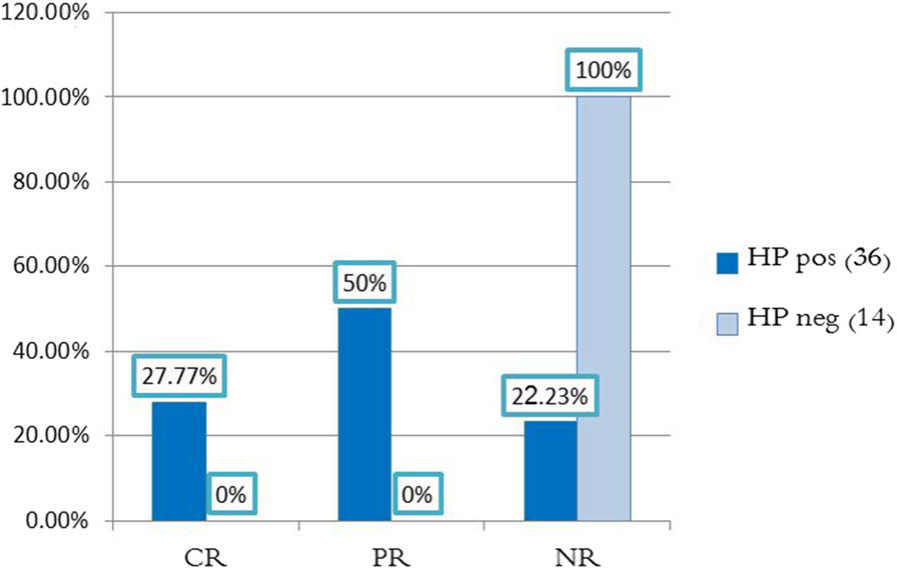
Comparison of the final response results (end of the sixth month) between the two groups of *H. pylori*

We conclude from Fig. 2 that: Although the mean baseline platelet count is greater in Hp^+^ group, the difference between Hp^+^ and Hp^−^ groups is getting greater in the subsequent follow-up visits, as indicated by the gradually increased space between the curves. All that confirms the significant difference of platelet counts between the two groups (Table 2).

**Fig. 4:**
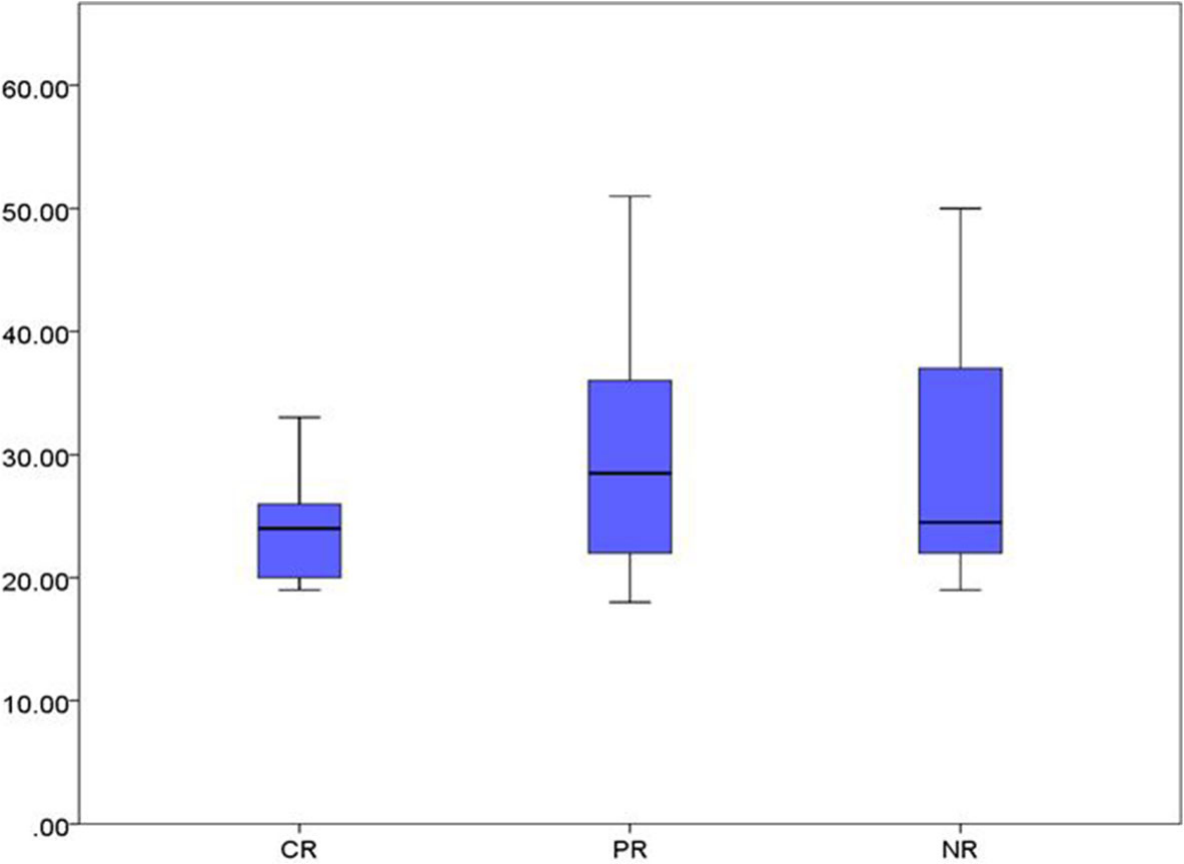
Comparison of median age between complete response CR and partial response PR and no- response NR

**Fig. 5:**
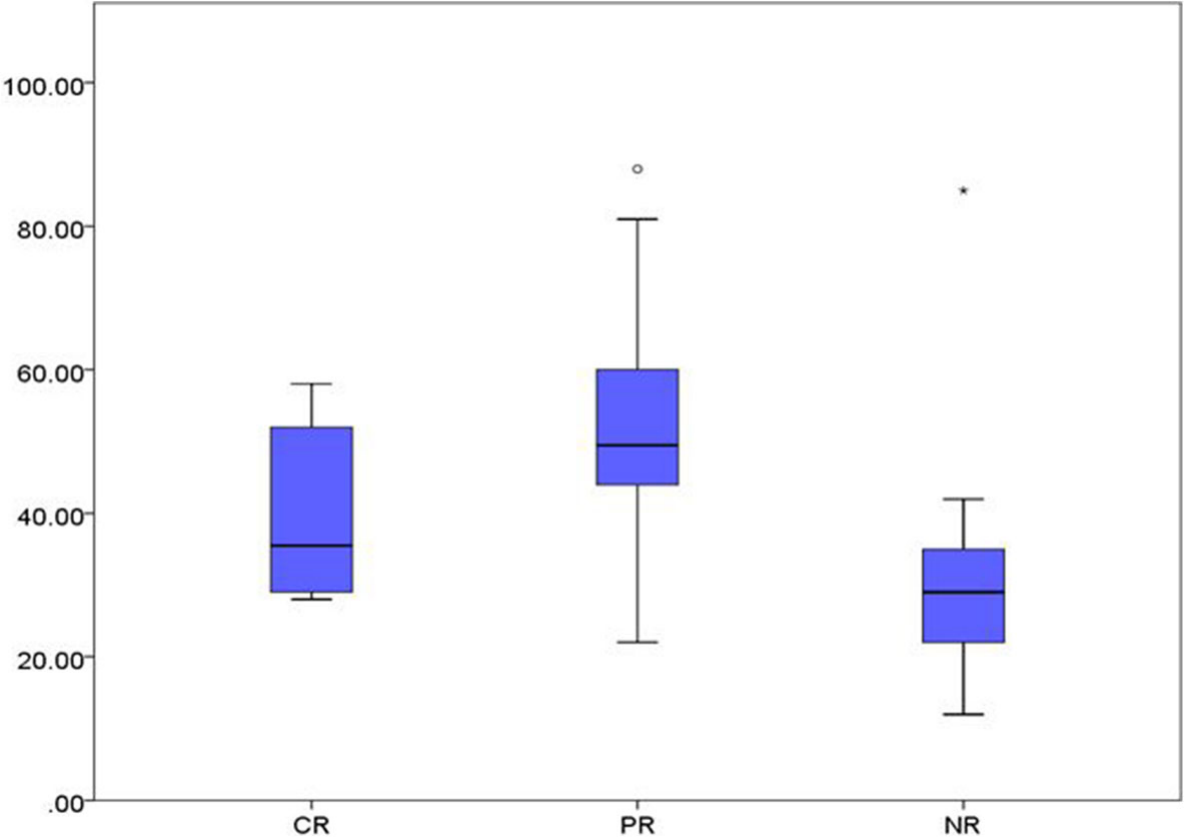
Comparison of the median value of platelet count at the start of the study between complete response cases CR and partial response PR and non-response NR

In conclusion, 10 Hp^+^ patients (27.77%) achieved complete remission, and 18 Hp^+^ patients (50%) achieved partial response. All responding patients are Hp^+^ (who received triple therapy for H.pylori). 8 Hp^+^ patients (22.23%) achieved no response. All the 14 (100%) Hp− patients (who received no treatment) did not achieve neither complete nor partial response (Fig. 3), even though some patients showed a slight increase in platelet count (Table 3). In other words, it is possible to say that treatment of *H. pylori* has effectively increased platelet count, and this improvement was not spontaneous.

**Fig. 6:**
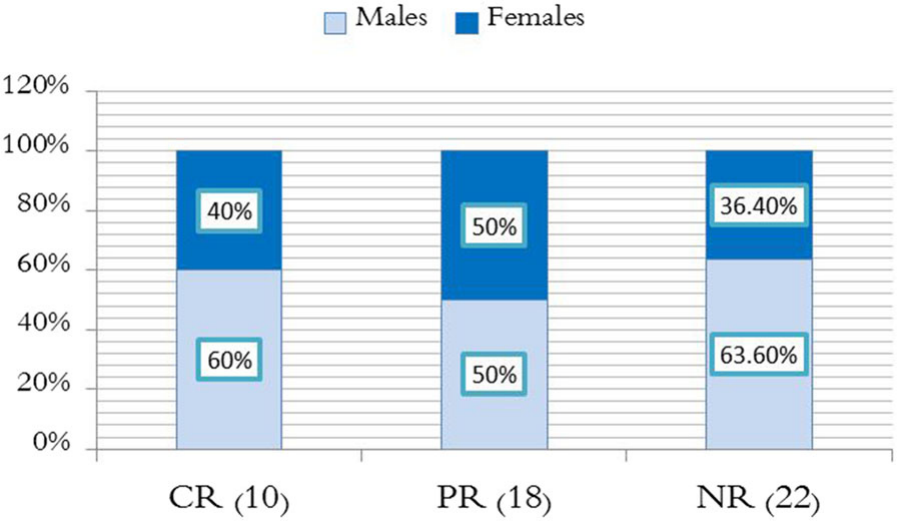
Comparison of sex distribution between complete response CR and partial response PR and non-response NR

**Table 3:** Comparison of the end result of the response (end of the sixth month) between the two groups of H. Pylori

	HP (+) (36)	HP (−) (14)	*P* value
Complete response	10 (27.77%)	0 (0%)	
Partial response	18 (50%)	0 (0%)	0.001
No response	8 (22.23%)	14 (100%)	0.001

### Comparison between complete response, partial response and no response groups

After obtaining the final results of patients at the end of the sixth month, they were divided into three groups according to the response:

- Complete response group, including 10 patients.
- Partial response group, including 18 patients.
- No-response group, including 22 patients.

We will compare the three groups in terms of age distribution, sex distribution, baseline mean platelet count, H.pylori infection status.

### First: Comparison of the mean age

The mean age of complete response group, partial response group, and no response group is 24.40, 28.95 years and 30.50 years, respectively as shown in (Fig. 4). One way ANOVA shows no statistically significant difference between these values. This means that age is not an effective factor in response Table 4.

**Fig. 7:**
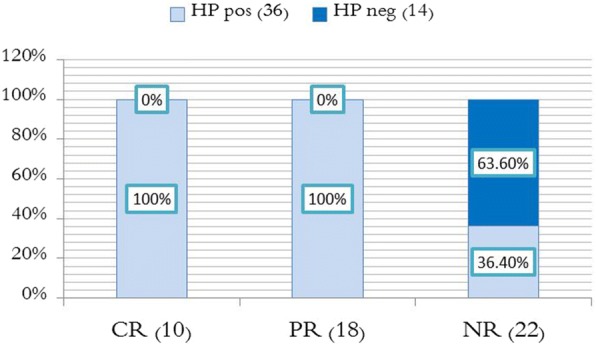
Comparison of the percentages of the *H. pylori* infection with complete response CR and partial response PR and non-response NR

**Table 4:** Comparison of the platelet count, and the patient’s sex, and the median age, by type of response

		Platelet count	Males	Females	Median age
Complete response	HP(+) (36)	40.50	6 (60%)	4 (40%)	24.40
	HP(−) (14)				
Partial response	HP(+) (36)	52.44	9 (50%)	9 (50%)	28.95
	HP(−) (14)				
No response	HP(+) (36)	30.41	14 (63.6%)	8 (36.4%)	30.50
	HP(−) (14)				

### Second: Comparison of the primary platelet count

The mean value of baseline platelet count in complete response group, partial response group and no response group is 40.50 × 10^9^/L, 52. × 10^9^/L and 30.41 × 10^9^/L, respectively as shown in (Fig. 5). One way ANOVA test indicates a statistical significance of these differences as shown in (p) Table 4.

### Third: Comparison of sex distribution

The complete response group included 6 males (60%) and 4 females (40%). The partial response group included 9 males (50%) and 9 females (50%). No response group included 14 males (63.6%) and 8 females (36.4%) as shown in (Fig. 6). Fisher Exact test indicates that there is no statistically significant difference between these ratios. Therefore, patient’s sex was not an effective factor to achieve response. As shown in Table 4.

### Fourth: Comparison of *H. pylori* infection status

All cases of complete and partial response were Hp^+^. Out of the 22 cases of the no response group, 8 patients (36.4%) were Hp^+^, and 14 patients (63.6%) were Hp^−^ as shown in (Fig. 7). Chi-square test indicates a statistically significant difference between these groups in H.pylori infection status. In other words, the response cases were more common among the HP (+) patients. That means *H. pylori* eradication increased platelet count, because no other treatment was given. As shown in Table 5.

**Table 5:** Comparison the percentages of the H. Pylori infection by type of response

Group	HP (+) (36)	HP (−) (14)	*P* value
Complete response(10)	10 (100%)	0 (0%)	Less than 0.001
Partial response (18)	18 (100%)	0 (0%)	
No-response (22)	8 (36.4%)	14 (63.6)	

## Discussion

We gave Triple therapy to Hp^+^ patients with no additional treatment, and Hp^−^ were monitored without any treatment. Mean baseline platelet count was significantly higher in Hp^+^ group. Mean platelet count of Hp^+^ patients was also significantly higher than that of Hp^−^ at the end of the first, third and sixth month. Mean platelet count markedly improved in Hp^+^ group, and the difference in mean platelet count was gradually getting greater in subsequent follow- up visits at the end of the first month and third month.

We notice a slight decrease in the mean platelet count of Hp^+^ group at the end of the sixth month compared to that of the third month, so do the difference in means between Hp^+^ and Hp^−^ groups. But the mean platelet count of Hp^+^ group at the end of the sixth month is still greater than that at the beginning of the study and at the end of the first month, so do the difference in means between Hp^+^ and Hp^−^ groups. Mean platelet count in Hp^+^ group at the end of the sixth month is still greater than the mean platelet count of Hp^−^ group at all stages.

The difference between mean baseline platelet count and mean platelet count at the end of the sixth month was significantly higher in Hp^+^ group.

Patients who achieved complete or partial response were all Hp^+^, while Hp^−^ patients did not achieve neither complete nor partial response. The previous findings indicate that the improvement noticed in Hp^+^ group is often due to H.pylri eradication, not a spontaneous improvement.

Age and sex are not related to achieving response, while baseline platelet count and H.pylori infection status do.

### Scientific explanation of this improvement

The pathogenic role of *H. pylori* infection in the development of ITP is still unclear, several mechanisms are suggested: *H. pylori* may modify the balance of Fc-receptors on the surface of monocytes. This modification leads to activation of these receptors, which makes monocytes attack platelets. Bacterial antigens and glycoprotein platelet surface antigens express some similarity. When antibodies are produced against these bacterial antigens, they interact with the glycoprotein Platelet surface antigens, thus lead to decrease platelet count. Some strains of *H. pylori* induce platelet activation mediated by *H. pylori*-bound vWF interacting with GPIb, and supported by IgG. [11–15].

Study limitations include the small sample size due to the rarity of ITP. Another limitation is the absence of a control group consisted of healthy asymptomatic participants to compare the prevalence of H.pylori infection between ITP patients and the healthy population.

## Conclusion

- *H. pylori* eradication in ITP patients Leads to significant Improvement of peripheral blood platelet count in most patients.

### Recommendations

- To investigate for *H. pylori* infection in ITP patients, even in the absence of gastrointestinal symptoms.
- To consider the triple anti-H.pylori therapy as a first therapeutic option for Hp^+^ ITP patients, with periodic monthly monitoring of platelet count during the first 6 months to confirm the response.
- To conduct a large-scale well-controlled case-control studies to confirm the relationship between H.pylori infection and the development of ITP.
- To conduct randomized controlled trials to confirm the benefit of H.pylori eradication in Hp^+^ ITP patients.
- To conduct a systematic review and meta-analysis of the available literature.

## Abbreviations

*H. pylori*: Helicobacter pylori
HCV: hepatitis C virus
HIV: human immunodeficiency virus
HP: Helicobacter pylori
ITP: Idiopathic Thrombocytopenic Purpura
IVIG: Intravenous immunoglobulin
SLE: Systemic lupus erythematous
SPSS: a statistical package for the social sciences
TTP: thrombotic thrombocytopenic purpura
