# Correction to: The impact of helicobacter pylori eradication on platelet counts of adult patients with idiopathic thrombocytopenic purpura

**DOI:** 10.1186/s12878-019-0142-7

**Published:** 2019-05-30

**Authors:** Sara Aljarad, Ahmad Alhamid, Ahmad Sankari Tarabishi, Ameen Suliman, Ziad Aljarad

**Affiliations:** 1 Department of Hematology, Al Mouwasat University Hospital, Damascus, Syria; 2 Department of Gastroenterology, Aleppo University Hospital, Aleppo, Syria; 3 Medical student, Faculty of Medicine, University of Aleppo, Aleppo, Syria

**Correction to:** **BMC Hematol (2018) 18: 28**

**https://doi.org/10.1186/s12878-018-0119-y**

In the original version of this article [1], published on 20 September 2018, there was an error in the author name of Dr. Sankari Tarabishi.

Originally the author name was published/cited as:

Tarabishi, AS.

The correct name is:

Sankari Tarabishi, A.

The correct name and citation is available via this correction article.
